# A “Proof of Concept” Randomized Controlled Trial of a Video Game Requiring Emotional Regulation to Augment Anger Control Training

**DOI:** 10.3389/fpsyt.2021.591906

**Published:** 2021-09-01

**Authors:** Peter Ducharme, Jason Kahn, Carrie Vaudreuil, Michaela Gusman, Deborah Waber, Abigail Ross, Alexander Rotenberg, Ashley Rober, Kara Kimball, Alyssa L. Peechatka, Joseph Gonzalez-Heydrich

**Affiliations:** ^1^Department of Psychiatry, Boston Children's Hospital, Boston, MA, United States; ^2^Department of Psychiatry, Harvard Medical School, Boston, MA, United States; ^3^Neuromotion Labs, Boston, MA, United States; ^4^Department of Psychiatry, Massachusetts General Hospital, Boston, MA, United States; ^5^Department of Neurology, Boston Children's Hospital, Boston, MA, United States; ^6^Department of Neurology, Harvard Medical School, Boston, MA, United States

**Keywords:** anger control, biofeedback, video game, cognitive behavior therapy, emotional control, self-regulation

## Abstract

Emotional dysregulation leading to clinically significant anger and aggression is a common and substantial concern for youth and their families. While psychotropic medications and cognitive behavioral therapies can be effective, these modalities suffer from drawbacks such as significant side effects, high rates of attrition, and lack of real-world skill translation. **R**egulate **a**nd **G**ain **E**motional Control (RAGE-Control) is a video game designed as an engaging augment to existing treatments. The game facilitates emotional regulation skill building through practice modulating physiological arousal while completing a challenging inhibitory task. We compared reduction in anger, aggression, oppositionality, and global severity between two treatment conditions: Anger Control Training (ACT) augmented with RAGE-Control and ACT with a sham version of the game, in a pilot double-blind randomized controlled trial. To begin to understand mechanisms of change, we examined heart rate during game play over the course of the study and explored associations between symptom changes and heart rate changes.

**Materials and Methods:** Forty youth with clinically significant anger dyscontrol (age 10–17) were randomly assigned to 10 sessions of ACT with RAGE-Control or ACT with sham video game.

**Results:** Both treatments similarly reduced self-reported anger. However, ACT with RAGE-Control led to larger improvements in aggression (CI: −17 to −1.0, ES: 0.55, *p* = 0.015); oppositionality (CI: −9.0 to −7e-6, ES: 0.48, *p* = 0.032); and global severity (CI: −1.0 to −5e-6, ES: 0.51, *p* = 0.023) relative to sham. Participants in the RAGE-Control group saw a decrease in median heart rate during game play (β = 1.2, *p* < 0.001). Larger pre to post decreases in heart rate were significantly associated with larger pre to post decreases in aggression and oppositional behaviors.

**Discussion:** Augmenting ACT with RAGE-Control reduced behavioral expression of anger, but not the experience of angry feelings, as compared to ACT with a sham version of the game. Increased heart rate control, demonstrated by reduction in median heart rate during gameplay, was associated with decreased aggression and oppositional behavior. Together these findings support that augmenting traditional treatment with technology facilitating heart rate control through skill practice translates to enhancements in real-life behavioral change. Therefore, further exploration into engaging skill-focused games such as RAGE-Control is warranted.

**Clinical Trial Registration:**ClinicalTrials.gov, identifier: NCT01551732.

## Introduction

### Why Build a Video Game Targeting Emotional Dysregulation?

Emotional dysregulation, defined as a limited ability to initiate and regulate one's emotional reaction and response in a manner consistent with the situation (1), is a major challenge for children and adolescents who struggle with anger and aggression (2–4). Emotional dysregulation is also a common feature of behavioral health and neurodevelopmental disorders including ADHD, Autism Spectrum Disorders, oppositional defiant disorder, and others (5–7). Irritability, an increase studied facet of emotional dysregulation, is becoming transdiagnostic hallmark of child and adolescent psychopathology (8). Several evidence-based treatments including medication, cognitive behavioral therapies (CBT), and parent management training have been developed to address emotional dysregulation, anger, and aggression in youth (2, 9, 10). However, each modality suffers from downsides. Medication, despite moderate to large effect sizes in second-generation antipsychotics, is often considered a last resort due to significant side effects (11, 12). CBT and family/parent-based interventions have relatively moderate effect and suffer from high rates of attrition (2, 9, 10). Some potential pitfalls of psychotherapy for youth with emotional dysregulation include the lack of focus on problem-solving skills or experiential practice *outside* of therapy (13, 14), and the heavy focus on parental monitoring, which might elicit negative side-effects among adolescents in particular (15, 16). These challenges are particularly relevant to emotional regulation, as autonomy in practicing problem solving and reducing physiological arousal may promote youth's emotional development (17). The aim of building a video game for developing emotional regulation was to create an engaging, challenging augment to CBT to address the lack of outside practice and translatable application of emotional regulation skills.

Interest and development of video games, mobile applications, and other technology-based interventions, often called serious games, has grown over the past decade. In part, these interventions are attractive because they are easily accessible and provide an extension of clinical settings (18). Serious games also benefit from having high intensity, immediate reinforcement of learning objectives (19). Serious games have diverse applications, ranging from physical (20) to cognitive and social emotional domains (21–33) with both educational (34) and remedial (35) purpose. Games targeting regulation are examples of remedial social emotional interventions.

Studies on many emotional regulation-focused games have shown promising results, including games dedicated to development of emotion regulation skills (e.g., GameTeen) (23, 24), practicing intrapersonal or interpersonal responses to facilitate emotional intelligence (e.g., Spock) (25), enhancing rational thinking (e.g., REThink) (26), and reducing stress/anxiety [e.g., Dojo; (27) Mindlight; (28) Breathify (29)]. While much of this work has targeted adult populations (30–33), the positive impact of serious games for emotional regulation extends to adolescent populations as well (23–28). Perhaps one of the most well-studied serious games is PlayMancer, a bioresponsive game where a player's emotional state is measured using galvanic skin response, oxygen saturation, heart rate, and heart rate variability (30). Using PlayMancer has been associated with increases in self-report and physiological indicators of emotional regulation in individuals with eating disorders (31, 32) and severe gambling disorder (33).

### Why Focus on Heart Rate?

RAGE-Control (**R**egulate **a**nd **G**ain **E**motional Control) is a space-themed, non-violent video game that uses a player's heart rate to help them practice modulating physiological arousal while completing a challenging inhibitory task. In the game, the player is asked to “shoot” asteroids while allowing friendly craft to pass. Should the player's heart rate increase, they become unable to “shoot” the asteroids. Thus, players are rewarded for down-regulating physiological arousal (36, 37). As mentioned above, RAGE-Control was created to facilitate experiential learning and practice of emotional regulation. In order to successfully translate to emotional regulation, RAGE-Control operates under two overarching hypotheses. First, that children and adolescents are motivated and challenged enough by gameplay to practice skills learned in therapy to regulate their heat rate. Second, that greater control over one's physiological arousal, measured here by heart rate, can lead to greater emotional regulation.

While heart rate is a crude signal of regulation, a robust set of existing literature demonstrates a link between parasympathetic control over heart rate and self-regulatory capacity; (38–40) and parasympathetic control over heart rate and emotional regulation (41, 42). This work extends to behaviors and diagnostic categories associated with poor emotional regulation in children and adolescents, as high heart rate reactivity in response to stressors is associated with externalizing behaviors (43–45) and aggression (46). Furthermore, children with conduct disorder have greater heart rate reactivity to frustration as compared to those without (47). This study, and the creation of RAGE-Control, proposes that heart rate regulation in the moment of difficult or demanding situations is a clinically useful translation of existing theory.

### “Proof of Concept” Randomized Controlled Trial

The objective of this double-blind randomized controlled trial (RCT) is to provide an initial assessment of clinical benefits from incorporating RAGE-Control into Anger Control Therapy (ACT), an empirically supported, manualized CBT for anger control (48, 49). A large body of evidence suggests that interventions in the form of video games are a well-accepted and clinically impactful area of study (21–33), however, most widely available digital interventions do not have evidence from rigorous participant and clinician blinded RCTs (50, 51). In this study, children and adolescents with clinically significant anger problems were randomized to either ACT augmented with RAGE-Control at the end of each session, or ACT with a sham version of RAGE-Control. We hypothesized that participants assigned to the ACT with active RAGE-Control group would have greater decreases in anger, oppositionality, overt aggression, and clinician rated global severity as compared to those assigned to the ACT with sham group.

## Materials and Methods

### Patients

Patients between 10 and 17 years of age were recruited from the outpatient psychiatry clinic at Boston Children's Hospital (BCH) between July 2011 and February 2013. Patients were referred to the study if they experienced symptoms consistent with clinically impairing anger or aggression.

Inclusion criteria were age, clinician referral, and elevated self-reported anger confirmed by a score of ≥15 on the Trait Anger subscale of the State Trait Anger Expression Inventory-Child and Adolescent version (STAXI-CA) (52). Exclusion criteria were change in psychotropic medication dose within the 4 weeks prior to enrollment, anticipated change in psychotropic medication dose throughout the study period, or DSM-IV-TR diagnosis of intellectual disability.

Prior to enrollment, patients attended a screening visit where a licensed clinical social worker performed a mental health evaluation, reviewed available past records, and assigned a best estimate primary DSM-IV-TR diagnosis using a DSM-IV-TR symptom checklist (53). Parents provided information necessary to complete the Modified Overt Aggression Scale (MOAS) and Disruptive Behavior Rating Scale (DBDRS) for the patient based on the month before entering the study (54–56).

**Figure 1:**
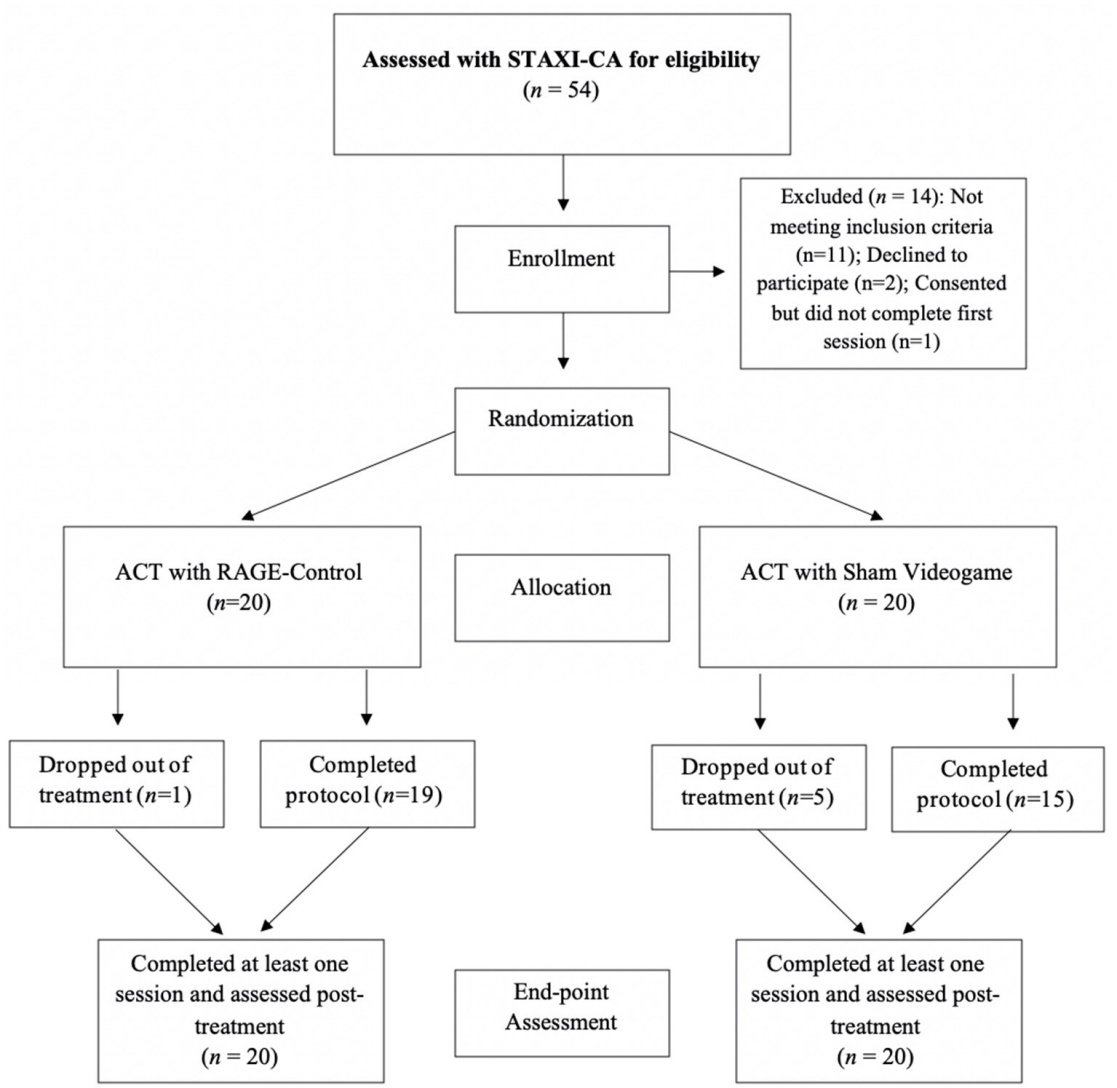
Participant recruitment and study flow.

Fifty-four children and adolescents were screened and 40 were enrolled/randomized (*n* = 20 in each group; Figure 1). Sample size was determined by power analysis, targeting 80% power to detect effect sizes (ES = 0.63–1.68) from a prior, preliminary, open-label study (57). All procedures contributing to this work comply with international ethical standards on human experimentation including the Helsinki Declaration of 1975, as revised in 2008 and were approved by the BCH institutional review board (IRB-P00000440). Written informed consent was obtained from a parent or legal guardian of all participating patients. Verbal assent was also obtained from patients and formally recorded. Families received $125 and complimentary parking for participation.

### Study Design

Immediately following screening and enrollment, patients were randomized into two groups: ACT augmented with RAGE-Control (ACT-R) or ACT augmented with a sham version of RAGE-Control (ACT-S; see below for details). Research staff generated the randomization sequence, enrolled patients, and assigned patients to treatment groups. Clinicians, families, and patients were blinded to group assignments throughout the study. Patients completed 10 weeks of ACT-R or ACT-S followed by a final study visit occurring 2 weeks post-treatment. To minimize data loss and bias associated with early termination, parents or guardians agreed to return for the post-treatment visit even if the intervention was terminated early.

The primary outcome measure for this study was the STAXI-CA. Patients completed this self-report measure at the screening visit, every other week at ACT sessions, and at the post-treatment visit. Secondary measures included standard assessments of aggression (Modified Overt Aggression Scale; MOAS), oppositionality (Disruptive Behavior Rating Disorder Scale; DBDRS), and global severity/improvement (Clinical Global Impressions Severity/Improvement; CGI-S and CGI-I, respectively). These measures were completed by parents (MOAS and DBDRS) or blinded clinicians (CGI) at screening and post-treatment visits only. Each time the patient played their assigned version of RAGE-Control, the device recorded their heart rate, allowing us to calculate median heart rate during gameplay.

### Interventions

#### RAGE-Control Video game

RAGE-Control is loosely based on the arcade game *Space Invaders*, requiring players to maneuver a spaceship at the bottom of the screen to fire at enemy spaceships while inhibiting fire as friendly spaceships fly past. During gameplay, players must control their heart rate, measured by pulse oximeter, to allow their spaceship to fire. That is, if a player's heart rate exceeds baseline by 7 bpm, their spaceship will fire blanks. These blanks do not destroy the asteroids and are accompanied by a different sound, indicating that the player needs to regulate their heart rate. Baseline heart rate was measured at the beginning of each play session while players sat quietly for 30 s.

Each round of RAGE-Control is 3 min long and patients played several rounds at the end of each session, followed by discussion about what worked well to lower their heart rate.

##### Active vs. Sham Game Condition

The active (ACT-R) and sham (ACT-S) versions of the RAGE-Control game were identical, including wearing a heart rate monitor collecting heart rate data, except in the sham condition the player's heart rate was not an input into the game. As a result, patients in the ACT-S condition did not need to control their heart rate for their spaceship to function.

#### Anger Control Therapy

Each patient was assigned to one of two research therapists (licensed clinical social workers) based on scheduling convenience. The lead therapist (first author) trained the second study therapist in a 2-h initial training and then met for weekly supervision during the study. Both the ACT-R and the ACT-S groups engaged in 10 h-long ACT sessions once a week.

During the first five sessions of ACT, patients learned a specified coping skill to regulate their mood and behavior; they subsequently spent 15 min playing either the real or sham version of RAGE-Control, during which they were instructed to use the coping skills learned. A parent check-in took place at the end of each session that ranged from 5 to 15 min to discuss how the child was doing from the parent's perspective.

Sessions 6–10 involved structured problem solving on applying coping skills to real-life current problems and continued practice with the assigned version of RAGE-Control. A parent check-in also took place at the end of the sessions 6–10 that ranged from 5 to 15 min to review problems and have patients teach their parents the coping/relaxation skills they have learned by showing them how to play RAGE-Control and playing together as a team.

##### Treatment Fidelity

Sessions were delivered using a detailed manual written by the first author, P.D., as an adaptation of Anger Control Training by Sukhodolsky et al. (49) This is the first study using this manual. We have made the manual available at the following web address: https://drive.google.com/open?id=0BwtznSVw1ibUSndUMV9YdnROZGc. Sessions were audio recorded and fidelity checklists were used to document implementation of the specific ACT goals for each session (58). Twenty percent of the recorded sessions were selected at random and rated by an independent social work intern using the fidelity checklist. A high level of treatment fidelity was demonstrated (93% mean; range 81–100%).

### Outcome Measures

#### State Trait Anger Expression Inventory—Children and Adolescents Trait Anger Subscale

This 35-item scale measures self-reported feelings of anger. The STAXI-CA has good construct validity and internal consistency with Cronbach's alpha coefficients range from 0.86 to 0.93 (52). It was administered at baseline and at the end of every other treatment session. Because it measures how often and intensely angry feelings are experienced over time, the STAXI-CA-Trait Anger (TA) subscale was chosen to measure levels of self-reported anger symptoms.

#### Modified Overt Aggression Scale

This 5-point scale rates the severity of four types of aggression: verbal, against property, against self (auto-aggression), and physical toward others (54). Each of these four subscores contains five different levels that contribute to the score. These individual levels are differentially weighted to discriminate behavior in increasing severity (e.g., for verbal aggression, “Shouts angrily” is weighted as 1 and “Threatens violence toward others” is weighted as 4). Each subscore is calculated by adding up the weights for each level present. In total, each of these subscores has a range of 0–10. The subscores are further weighted in the calculation of total score by multiplying each by a number representing its relative severity (e.g., one for verbal aggression and increasing to a multiplier of 4 for aggression against others). The weighted subscores are then added to get the total score. A copy of the scale can be found at https://depts.washington.edu/dbpeds/Screening%20Tools/Modified-Overt-Aggression-Scale-MOAS.pdf.

The assessor read each item of the MOAS to the parent, including the examples of behaviors anchoring each potential scoring statement, asked whether each statement describes the child's behavior over the previous week, and noted the parent's response.

#### Disruptive Behavior Disorders Rating Scale

This 8-item scale evaluates symptoms of Oppositional Defiant Disorder (ODD). It rates the parent's perspective of their child's oppositional behavior in four domains (1) degree of symptom presence, (2) level of concern/interference with daily activities, (3) level of monitoring required, and (4) level of attention required. Each item is rated on a 4-point Likert scale ranging from 0 = never or rarely to 3 = very often and derives a total score by summing all items together. Internal consistency ranges from 0.86 to 0.93 (55, 56).

The assessor read the parent each question of the DBDRS and asked the parent to indicate the degree to which each statement describes the child's behavior in the past week (“not at all,” “just a little,” “pretty much,” and “very much”). The MOAS and DBDRS thus assessed the parent's perspective of their child's level of aggression and oppositionality in the previous week.

#### Clinical Global Impression-Severity and Improvement Scale

The CGI-S scale requires a clinician to rate the overall severity of psychopathology on a 7-point Likert scale, ranging from 1 (normal, not ill) to 7 (extremely ill). The CGI-I requires the clinician to rate total improvement whether or not, in the raters judgment, it is due to treatment. The clinician compared the patient's condition at baseline to 2 weeks after the study treatment ended on a scale ranging from 1 (very much improved) to 7 (very much worse) (59).

Clinician-parent interviews were audiotaped and an independent assessor re-rated a randomly selected 20% of parent interviews to establish inter-rater reliability for the MOAS, DBDRS, CGI-S, and CGI-I measures. A weighted Cohen's *K* documented acceptable inter-rater reliability in the study for the MOAS (0.92 at baseline; 0.88 at post treatment), DBDRS (0.91 at baseline; 0.83 at post treatment), CGI-S (0.77 at baseline, 0.82 post treatment) and CGI-I (0.83 at post treatment).

#### Median Heart Rate

Heart rate was captured at 1 Hz intervals using a pulse oximeter during gameplay in both ACT-S and ACT-R conditions. The median heart rate during gameplay for each patient at each session was calculated.

### Statistical Methods

To reduce bias resulting from drop-out, statistical analyses were conducted using an intent to treat methodology. Therefore, data from patients who completed at least one treatment session were included in the analysis. Per protocol, all parents of participants who entered the study provided data at the post-intervention timepoint. *T*-tests and Fisher's exact tests were used to compare demographic characteristics and retention/early dropout rates. Pre-post treatment changes in outcome measures were compared between the groups using Wilcoxon rank-sum tests.

For the STAXI-CA-TA subscale, a mixed linear regression model with fixed effects of session, group, and their interaction, and random effects for participants was used to estimate change over time. The treatment group by time interaction term in this model was examined as an indicator of whether one treatment was more efficacious in decreasing frequency of angry feelings than the other. To account for multiple comparisons, we used a false discovery rate method (FDR) (60) as recommended for health studies when the study endpoints are interdependent with each other and not independent as assumed in a Bonferonni correction (61). The procedure employed a tail-based false discovery rate that takes as its input the two-tailed *p*-values obtained from the multiple hypotheses tests (62, 63). We report an adjusted *p*-value for each comparison.

Changes in median heart rate during game play were explored with a linear mixed effects model with fixed effects of session, group, and their interaction, and random effects for participants. Exploratory relationships between change in symptoms and change in median heart rate were examined by calculating post-pre change scores for all variables and employing bivariate Spearman's correlations.

All hypotheses were accepted at a two tailed significance level of α = 0.05.

## Results

### Patient Characteristics

Table 1 demonstrates that the groups were comparable in baseline demographic and clinical characteristics and describes the diagnoses assigned at the screening visit to each patient.

**Table 1:** Demographic and clinical characteristics of treatment groups.

	**ACT-R (*n* = 20)**	**ACT-S (*n* = 20)**	***t***	***p***
				***t*-test**
Age mean (std) min, max	13.1 (2.4) 10.0, 17.0	12.4 (2.1) 10.0, 17.0	0.98	0.31
School grade mean (std) min, max	7.8 (2.6) 4.0, 11.0	6.7 (2.3) 4.0,12.0	1.42	0.16
				**Fisher's exact**
Male	14 (70%)	15 (75%)		1.0
Black	3 (15%)	6 (30%)		0.45
White Non-Hispanic	13 (65%)	8 (40%)		0.20
Hispanic	4 (20%)	6 (30%)		0.72
Father at home	3 (15%)	6 (30%)		0.45
Medication^*^	4(20%)	5 (25%)		1.0
**Baseline ratings**				
**Rating scale**	**Median (interquartile range)**		**W**	**Wilcoxon rank-sum test**
MOAS	18 (18.75)	5 (19.5)	173.5	0.48
DBDRS	17 (7.75)	17 (9.25)	187.5	0.74
CGI-S	5 (1)	4 (1)	165.5	0.32
STAXI-CA-TA	21 (8)	21 (5)	198	0.97
**Clinician Assigned Best Estimate Primary DSM-IV TR Diagnoses**				
**Participant DSM-IV diagnosis**		**ACT-R**		**ACT-S**
Opposition defiant disorder (ODD)		13		10
Attention deficit hyperactivity disorder (ADHD)		3		5
Major depressive disorder (MDD)		2		2
Generalized anxiety disorder (GAD)		1		1
Post traumatic stress disorder (PTSD)		1		
Depressive disorder NOS				1
Anxiety disorder (NOS)				1

* *Five (25%) patients on the ACT-R arm were on medications (n = 2 stimulant, n = 1 SSRI, n = 2 antipsychotic). Four (20%) patients on the ACT-S arm were taking medications (n = 1 stimulant; n = 1 antipsychotic; n = 1 stimulant, mood stabilizer and antipsychotic; n = 1 SSRI and mood stabilizer)*.

### Outcomes

Table 2 shows pre-post changes in outcome measures for the two groups and results of statistical tests.

**Table 2:** Pre-post treatment changes in outcomes.

	**ACT-R** ** (*n* = 20)**	**ACT-S** ** (*n* = 20)**				
	**Median change pre to post (interquartile range)**	**Median change pre to post (interquartile range)**	**Statistic**	**95% CI**	**ES**	***p***
MOAS	−8 (18.25)	0 (6.5)	W = 110	−17.0 to −1.0	0.55	0.015^*^
DBDRS	−7 (9)	0 (9.25)	W = 121	−9.0 to −7e-6	0.48	0.032^*^
CGI-severity	−1 (2)	0 (1)	W = 119	−1.0 to −5e-6	0.51	0.023^*^
	Median (interquartile range)	Median (interquartile range)				
CGI-I	2 (1)	3 (2)	W = 190	−3e-5 to 2.0	0.37	0.10
Early drop out rate	1/20	5/20		0.0–1.7	0.07	0.18

* *Statistically significant at p < 0.05; Bolded p-values indicates that the comparison remained statistically significant after accounting for multiple comparisons with FDR (62, 63)*.

#### Retention/Early Dropout Rate

One patient in the ACT-R group dropped out after the fifth session; five in the ACT-S group dropped out after the third to fifth session. All patients dropped out because they did not want to continue. This difference in dropout between ACT-R and ACT-S groups was not statistically significant (*p* = 0.18).

#### Patient Self-Ratings of Anger

The analysis reported in Table 3 indicates that patients in both groups had decreased feelings of anger on the STAXI-CA over the treatment period with no between-group differences.

**Table 3:** Change in STAXI-CA-TA.

**(A) FIXED EFFECTS**					
	**Estimate**	**df**	***t***	***p***	**5–95% CI**
Intercept (ACT-R group)	21.3 (±0.9)	63.2	23.2	<0.001	19.5–23.1
Session number	−0.6 (±0.08)	153.2	−7.5	<0.001	−0.8 to −0.5
ACT-S group (control)	−0.6 (±1.3)	63.2	−0.5	0.630	−3.2 to 1.9
Session number x ACT-S group	0.11 (±0.12)	154.7	1.0	0.340	−0.1 to 0.4
**(B) RANDOM EFFECTS BY PARTICIPANT**					
	**Parameters**	**AIC**		**df**	***p*** **(>chi square)**
No random effect	5	1,109			
Random effect by participant	6	1,042		1	<0.001

*A mixed model for change in STAXI-CA-TA ratings with **(A)** fixed effects for group, session, and for the interaction of group and session; and **(B)** random effects for participant. Analysis shows no interaction between group and session number such that group did not have an impact self-reported anger frequency over the course of the study*.

#### Parent Reported Aggression and Oppositional Behavior

Pre to post changes in parent reported aggression and behavior were significantly greater in the ACT-R group than the ACT-S group (Table 2).

#### Blinded Clinician Ratings

As shown in Table 2, patients in the ACT-R group showed significantly greater decreases in overt aggression (MOAS), disruptive behaviors (DBDRS), and global severity (CGI-S).

#### Median Heart Rate

**Figure 2:**
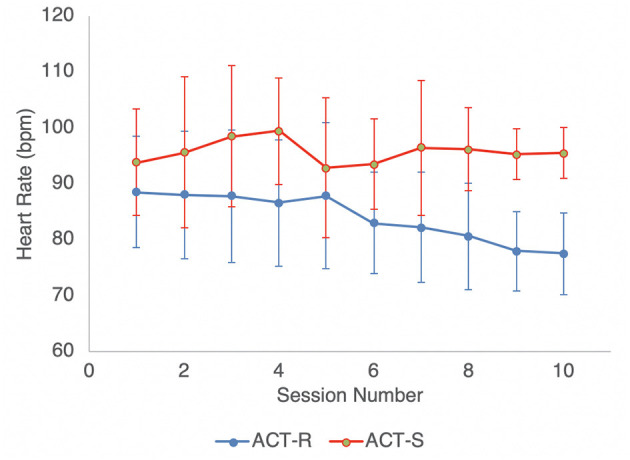
Median heart rate during gameplay by Session.

Median heart rate for each group at each session is displayed in Figure 2. Because keeping heart rate from becoming elevated during game play was an object of the active RAGE-Control game but not of the sham RAGE-Control game, a linear mixed effects model was used to test if there were group differences in the median heart rate during game play as the number of sessions increased. The analysis of the random effect of participant and fixed effects of session, group, and their interaction is displayed in Table 4. Median heart rate decreased each session for the ACT-R group, while it remained approximately constant across sessions for the ACT-S group (β = 1.2, *p* < 0.001).

**Table 4:** Change in heart rate.

**(A) FIXED EFFECTS**					
	**Estimate**	**df**	***t***	***p***	**5–95% CI**
Intercept	90.9 (±2.2)	46.1	41.6	<0.001	87.3–94.6
Session number	−1.1 (±0.2)	264.8	−7.4	<0.001	−1.4 to −0.9
ACT-S Group (Control)	4.9 (±3.3)	47.8	1.5	0.143	−0.6–10.4
Session number x ACT-S Group	1.2 (±0.3)	269.4	4.3	<0.001	0.7–1.6
**(B) RANDOM EFFECTS BY PARTICIPANT**					
	**Parameters**	**AIC**		**df**	***p*** **(>chi square)**
No random effect	6	2,028.2			
Random effect by participant	5	2,254.5		1	<0.001

*A mixed model for change in median heart rate during game play with **(A)** fixed effects for group, session, and for the interaction of group and session; and **(B)** random effects for participant. Analysis shows an interaction between group and session number such that the ACT-R but not the ACT-S patients have a decrease in median heart rate during game play as session number increases*.

Consistent with previous analyses, exploratory bivariate Spearman's correlations between STAXI-CA, MOAS, and DBDRS change scores revealed significant relationships between behavior change and heart rate, but not self-reported anger. Specifically, larger pre to post decreases in heart rate were significantly associated with larger pre to post decreases in aggression (MOAS; *r*_s_ = 0.43, *p* = 0.009) and oppositional behaviors (DBDRS; *r*_s_ = 0.38, *p* = 0.024), but not pre to post changes in self-reported anger (STAXI-CA; *r*_s_ = −0.28, *p* = 0.1).

## Discussion

This “proof-of-concept” pilot study demonstrates that ACT augmented with RAGE-Control yielded greater improvements in oppositional behavior, overt aggression, and clinician rated global severity as compared to ACT supplemented with a sham version of the game. However, the group receiving ACT augmented with RAGE-Control did not show greater decreases in self-reported levels of anger. This argues that augmenting ACT with RAGE-Control enhanced children's control of the expression of their anger rather than decreasing the frequency or intensity of their angry feelings.

### Implications for Treatment of Anger

While strongly related, the experience of anger and expression of anger are conceptualized as distinct (64). Anger is an affective state that includes increased physiological arousal and predisposition toward aggressive behavior whereas anger expression references the tendency to show anger outwardly, suppress it, or actively cope with the emotional experience (65). One explanation for the observed behavioral, rather than emotional, effect might be that skills introduced in ACT and reinforced in discussions about playing RAGE-Control were framed as skills to use when already experiencing strong emotions rather than to prevent experiencing the emotion itself. In fact, the initial studies examining the efficacy of ACT found a similar pattern of reduction in observed and self-reports of anger expression but not intensity of anger experiences (64, 66). In addition, RAGE-Control only indicates that a player's heart rate is high 1–2 s *after* it has happened. Therefore, the ACT-R group engaged in practice down-regulating their heart rate as a behavioral response, rather than preemptively working to maintain lower levels of arousal that might be interpreted as angry feelings.

### Relationship Between Heart Rate and Emotional Regulation

RAGE-Control was built on the premise that playing an engaging video game that rewards maintaining baseline heart rate would lead to increased heart rate control even in the face of in-game challenges. The present study supports that assertion, as the group playing the active version of RAGE-Control showed decreased median heart rate with gameplay, whereas median heart rate in the group who played the sham version remained approximately constant. Moreover, change in median heart rate was associated with change in reports of oppositional and aggressive behaviors, suggesting that ability to modulate heart rate is linked to maladaptive behavioral patterns in this population. While the sample size in this study prevents statistical analyses that would allow for more strong conclusions about causality or mechanisms of change, prior work demonstrating that parasympathetic heart rate control is *predicative* of self-regulatory capacity supports that interpretation (38–40).

Prior work utilizing HR biofeedback as a part of a gamified intervention called Playmancer failed to demonstrate reductions in HR across sessions, despite showing adaptive change in other physiological indicators of arousal (e.g., greater heart rate variability and lower respiration rate) (31). The inconsistency in these findings is likely a result of varied game goals. The focus of RAGE-Control is to explicitly down regulate HR, whereas goals for Playmancer were largely skill acquisition.

### Role of Context-Independence and Automaticity

One of the main criticisms of serious games is that such programs often do not have compelling evidence for generalizable, real world change (i.e., far-transfer effects) (21). Yet the current study suggests that playing RAGE-Control facilitated behavioral change noted by both blinded caregivers and blinded clinician raters outside of the game setting. A potential explanation for why RAGE-Control facilitates generalization is that RAGE-Control utilizes a different mechanism of learning than previous serious games. Rather than focus on application of skills in specific game contexts, RAGE-Control focuses on utilizing in game practice to establish an automatic response to internal stimuli (e.g., the player's heart rate). Not only does this mechanism allow for development of individualized skill, but it is also implicit, fast, and frequent during gameplay, facilitating automatic response learning. We propose that in combination, context-independence and repetition allow for far transfer effects (67). Future research would benefit the field by disentangling the relative contributions of context-independence and repetition.

### Potential Clinical Impact of RAGE-Control

One of the greatest advantages of incorporating games into youth therapy is the ability to engage individuals who might otherwise be hesitant to participate in treatment, including those with significant emotional dysregulation (68). In the current version of the Diagnostic and Statistical Manual of Mental Disorders (DSM-5), anger/irritability are core symptoms of Oppositional Defiant Disorder, Disruptive Mood Dysregulation Disorder, and aggression (anger expression) is the hallmark of Conduct Disorder. Furthermore, anger, irritability, and disruptive behaviors are commonly comorbid with other psychopathologies (69). In fact, 15 unique DSM-5 diagnoses identify some kind of emotional dysregulation as a symptom (70) and 7 DSM-4-TR diagnoses include irritable mood (53) (American Psychiatric Association, DSM4). The near ubiquity of emotional dysregulation in child psychopathology and high acceptability indicates that RAGE-Control could be a beneficial augment to cognitive-behavioral therapies with a wide range of patients.

Taken together these results are exciting because they demonstrate that (1) a video game can be used to practice heart rate regulation with skills taught in a therapeutic setting and (2) that such practice translates to behavioral and physiological change. This pilot study set a relatively high standard for proof-of-concept by way of a randomized design, equivalent therapist contact, exposure to the same CBT skills, and blinded raters. Additionally, the use of a sham computer game controlled for non-specific effects from the child playing a computer game at the end of each session. This pattern of results argues for further research to test the replicability of this study's findings, to understand if the results transfer to functional settings, and to clarify by what mechanism children randomized to the ACT-R condition are exerting better control of their angry behavior.

## Limitations

Despite rigorous design and promising findings, this proof-of-concept study has several limitations that warrant discussion. The first limitation is that the study includes a relatively small number of patients that restricts generalizability, inhibits exploration of age and sex differences, and necessitates replication of findings. The second limitation is a short follow up duration, as we did not assess patients further out than 2 weeks after completion of the intervention. Thus, these results do not provide information on how long observed behavioral improvements were sustained. Additionally, though the difference was not statistically significant, the ACT-S group had lower median MOAS score for the month prior to study entry than that ACT-R group (5 vs. 18, W = 173.5, *p* = 0.48), so that improvement in the ACT-S group may have been hampered by floor effects.

Early attrition from randomized control trials is a recognized problem that the study was designed to address. Specifically, all participants provided follow up data regardless of study completion. However, more patients in the ACT-S group discontinued treatment than in the ACT-R group. The decrease in early attrition for the ACT-R group did not reach statistical significance, however, the numerically greater early attrition from the ACT-S group raises the question of how much improvement in the ACT-R group was due to attending more ACT treatment sessions vs. practice of emotional regulation skills using RAGE-Control. Also, the median heart rate during the first game play session was lower for the ACT-R than for the ACT-S participants. This could have been because the ACT-R participants understood the game and were actively trying to control heart rate even during the first session; however, there could also have been failure of randomization and some unaccounted for physiologic difference between the groups may be responsible for the different trajectories of median heart rate over the 10 sessions. An additional limitation to this study is that it did not administer any adverse effect rating scale and thus did not systematically assess for adverse effects of the video games.

## Future Directions

Despite the failure of the groups to separate on the primary measure of frequency and intensity of angry feelings (STAXI-CA-TA), the consistent advantage of ACT-R over ACT-S on the secondary measures of oppositionality, overt aggression, and global severity in this proof of principle pilot study argue for further development of emotional regulation training games and further studies of their effects. Since the study reported here was completed, additional games requiring emotional control during game challenges have been developed and access to the games increased by porting them to mobile platforms such as smart phones and computer tablets (71). It will be important to determine if the greater variety and accessibility of these games improve their effectiveness in empowering parents to build their child's emotional regulation at home by providing more opportunity for practice. Additionally, providing an online forum where multiple people play together would also increase practice of emotional regulation skills and their generalization to social interactions. Given the difficulties parents experience in accessing child psychotherapists, enhancing these games with parent education modules should be studied to see if the games can be effective with little or no therapist contact. If successful, this would provide greater access for children and families in need of treatment for emotional regulation problems. Lastly, if the benefit of emotional regulation training video games is replicated in additional studies, more focused efforts to study the mechanisms of this benefit will be warranted. For example, ecological momentary assessment techniques could be employed at home along with heart monitoring to see if patient's actively controlling heart rate correlates with decreased aggressive and oppositional behavior in the home (72).

## Data Availability Statement

The raw data supporting the conclusions of this article will be made available by the authors, without undue reservation.

## Ethics Statement

The studies involving human participants were reviewed and approved by Boston Children's Hospital Institutional Review Board. Written informed consent to participate in this study was provided by the participants' legal guardian/next of kin. Participants also provided verbal assent that was formally documented.

## Author Contributions

PD, JK, and JG-H were integral in the study design, data collection, data analysis, and writing of this manuscript. DW contributed to study design and data analysis. ARot contributed to study design. CV, ARos, ARob, and KK were responsible for data collection. MG and AP were responsible for manuscript writing and preparation. All authors contributed to the article and approved the submitted version.

## Conflict of Interest

PD reports grants from the Deborah Munroe Noonan Memorial Research Foundation, Alrashed Family, and from Tommy Fuss Foundation, during the conduct of the study. JK reports grants from Noonan Foundation, during the conduct of the study. Boston Children's Hospital (BCH) owns the technology discussed in this paper. JK, PD, ARot, and JG-H founded and have equity in Neuromotion Labs, a company that creates emotional regulation training technologies, outside the submitted work. JK and AP report personal fees and other (equity) from Neuromotion Labs, outside the submitted work. In addition, ARot (along with JG-H, JK, and PD) have a pending patent US20140323013A1: Emotional control methods and apparatus licensed; In addition, JG-H has a patent European Patent 99112065.0-2201. Aug 17, 1999 US Patent 6,211,876. April 3, 2001 pending. CV reports Neuromotion Labs allowed her to use technology related to the RAGE Control videogame for free for another study that she was the Principal Investigator for. The remaining authors declare that the research was conducted in the absence of any commercial or financial relationships that could be construed as a potential conflict of interest.

## Publisher's Note

All claims expressed in this article are solely those of the authors and do not necessarily represent those of their affiliated organizations, or those of the publisher, the editors and the reviewers. Any product that may be evaluated in this article, or claim that may be made by its manufacturer, is not guaranteed or endorsed by the publisher.
